# Comparative genome analysis of three euplotid protists provides insights into the evolution of nanochromosomes in unicellular eukaryotic organisms

**DOI:** 10.1007/s42995-023-00175-0

**Published:** 2023-05-28

**Authors:** Didi Jin, Chao Li, Xiao Chen, Adam Byerly, Naomi A. Stover, Tengteng Zhang, Chen Shao, Yurui Wang

**Affiliations:** 1 Laboratory of Protozoological Biodiversity and Evolution in Wetland, College of Life Sciences, Shaanxi Normal University, Xi’an, 710119 China; 2 Institute of Evolution and Marine Biodiversity, Ocean University of China, Qingdao, 266003 China; 3 Laboratory of Marine Protozoan Biodiversity and Evolution, Marine College, Shandong University, Weihai, 264209 China; 4 Department of Computer Science and Information Systems, Bradley University, Peoria, 61625 USA; 5 Department of Biology, Bradley University, Peoria, 61625 USA

**Keywords:** Ciliate, *Euplotes*, Genome fragmentation, Gene family expansion

## Abstract

One of the most diverse clades of ciliated protozoa, the class Spirotrichea, displays a series of unique characters in terms of eukaryotic macronuclear (MAC) genome, including high fragmentation that produces nanochromosomes. However, the genomic diversity and evolution of nanochromosomes and gene families for spirotrich MAC genomes are poorly understood. In this study, we assemble the MAC genome of a representative euplotid (a new model organism in Spirotrichea) species, *Euplotes aediculatus*. Our results indicate that: (a) the MAC genome includes 35,465 contigs with a total length of 97.3 Mb and a contig N50 of 3.4 kb, and contains 13,145 complete nanochromosomes and 43,194 predicted genes, with the majority of these nanochromosomes containing tiny introns and harboring only one gene; (b) genomic comparisons between *E. aediculatus* and other reported spirotrichs indicate that average GC content and genome fragmentation levels exhibit interspecific variation, and chromosome breaking sites (CBSs) might be lost during evolution, resulting in the increase of multi-gene nanochromosome; (c) gene families associated with chitin metabolism and FoxO signaling pathway are expanded in *E. aediculatus*, suggesting their potential roles in environment adaptation and survival strategies of *E. aediculatus*; and (d) a programmed ribosomal frameshift (PRF) with a conservative motif 5′-AAATAR-3′ tends to occur in longer genes with more exons, and PRF genes play an important role in many cellular regulation processes.

**Supplementary Information:**

The online version contains supplementary material available at 10.1007/s42995-023-00175-0.

## Introduction

As one of the most diverse and specialized groups of single-celled eukaryotes, ciliated protists (ciliates) are featured by nuclear dimorphism (Fu et al. 2022; Lynn 2008; Zheng et al. 2021)—a germline micronucleus (MIC) that preserves all genetic information but remains transcriptionally inactive except during conjugation, and a somatic macronucleus (MAC) that is transcriptionally active and responsible for cell growth and reproduction (Sheng et al. 2020; Wei et al. 2022). During sexual conjugation, the MAC genome undergoes differentiation from a zygotic nucleus through genome-wide rearrangements such as chromosome fragmentation, micronuclear DNA elimination, and DNA amplification (Jahn and Klobutcher 2002; Zhang et al. 2022). Due to their unique features, ciliates have been important model organisms in cytology, genetics, environmental biology, and epigenetics (Sheng et al. 2021; Stoeck et al. 2018; Zhang et al. 2021).

According to genomic architecture, MAC genomes of ciliates can be divided into two broad categories (Maurer-Alcalá et al. 2018). One group consists of non-extensively fragmented chromosomes with “long” chromosomes (based on ciliates standards) carrying hundreds of genes. This group includes the class Heterotrichea (e.g., *Stentor*) (Chi et al. 2021), Oligohymenophorea (e.g., *Paramecium* and *Tetrahymena*) (Tian et al. 2022) and Colpodea (e.g., *Bursaria*). The other group has extensively fragmented chromosomes containing “gene-sized” chromosomes (termed “nanochromosomes”) capped with telomeres on both ends. This group includes the class Armophorea (e.g., *Metopus*), Phyllopharyngea (e.g., *Chilodonella*), Litostomatea (e.g., *Entodinium*), and Spirotrichea (e.g., *Euplotes*, *Oxytricha*, *Stylonychia*, *Pseudokeronopsis*, *Halteria*, and *Strombidium*) (Li et al. 2021; Mozzicafreddo et al. 2021; Swart et al. 2013; Zheng et al. 2021, 2022).

In the class Spirotrichea, nanochromosomes have an average length of 1–3.5 Kb and typically harbor only a single gene (Aeschlimann et al. 2014; Chen et al. 2019; Li et al. 2021; Swart et al. 2013; Vinogradov et al. 2012). Previous studies have primarily focused on genomic features such as chromosomal fragmentation, transcription regulation, programmed ribosomal frameshifting (PRF) events, genetic mechanisms of environmental stress response, and chromosome copy number (Aeschlimann et al. 2014; Chen et al. 2019; Swart et al. 2013; Zheng et al. 2022). However, due to high biodiversity of the Spirotrichea and limited genomic data available, the genomic diversity of MACs and the evolutionary history of nanochromosome fragmentation and gene function within this class remain poorly understood.

In this study, we characterize the nanochromosomes fragmentation patterns and PRF events in the MAC genome of *Euplotes aediculatus*. By combining our findings with genomic data from *E. vannus*, *E. octocarinatus* and other spirotrichs, we shed light on the hidden genomic diversity within *Euplotes* and Spirotrichea.

## Results

### Genome assembly of *Euplotes aediculatus* by deep sequencing

**Fig. 1:**
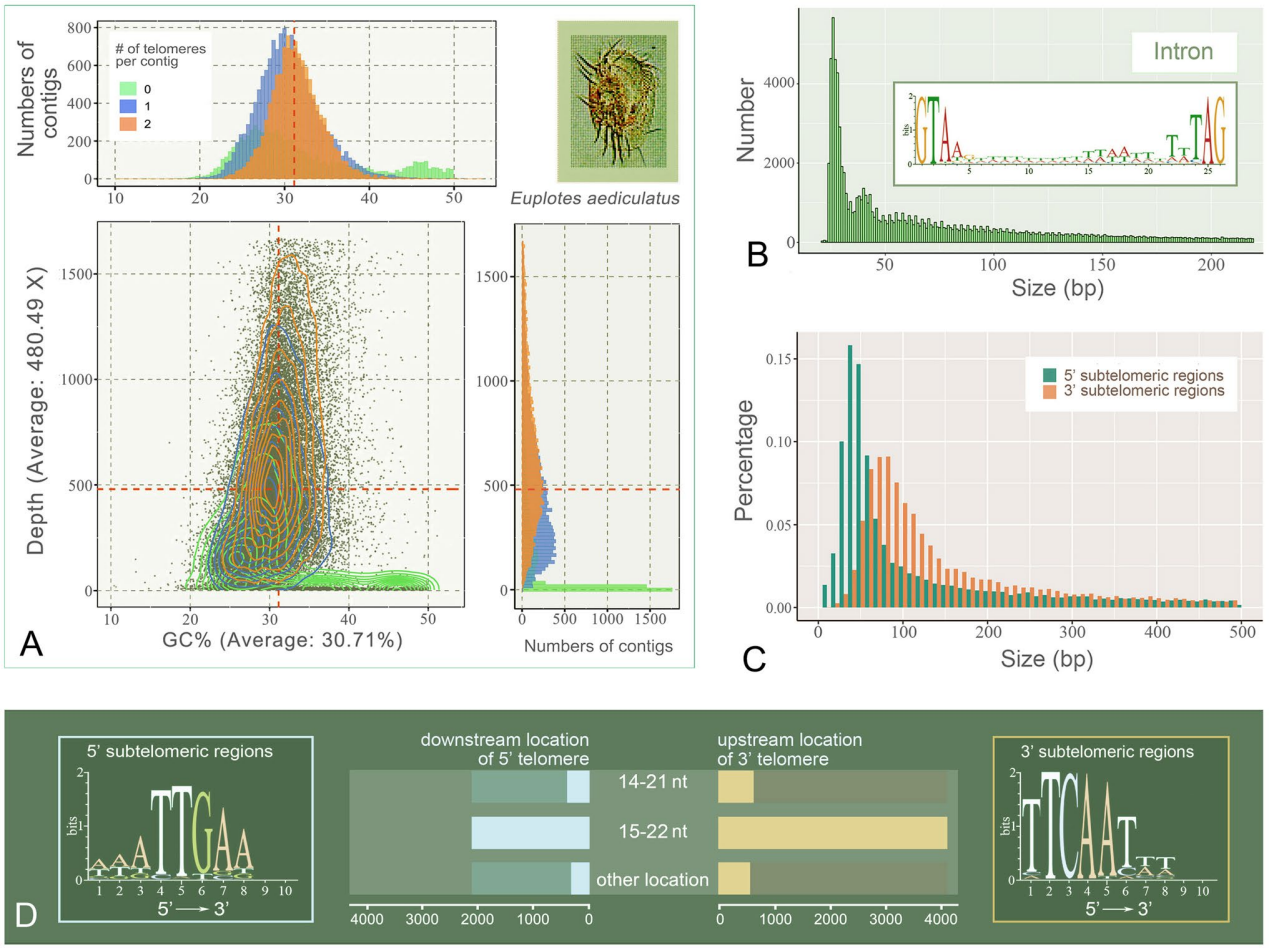
General features of the *E. aediculatus* genome assembly and chromosome structure. **A** The sequencing depth and GC content distribution of *E*. *aediculatus* contigs, grouped by telomere numbers. Each solid point represents a contig. Contour lines represent 2D density distribution of contigs. A ventral view of the species is shown in the top-right panel. **B** Size distribution of introns and sequence motif of tiny introns in the most abundant size category (5670 introns of 26 bp in length). The motif is flanked by canonical GT-AG splice site dinucleotides. **C** The length distribution of the 5′ and 3′ subtelomeric non-genic regions within 500 bp. **D** Two significant motifs (5′-WWATTGAA-3′ and 5′-TTCAATWW-3′) found in the 5′ and 3′ subtelomeric region, respectively. The location of motifs and frequency of location are shown in the middle part. Both motifs tend to be 14 nt away from telomeres

Deep sequencing revealed that *E. aediculatus* had a small and highly fragmented MAC genome with an average coverage of 480.49 × (Table 1). Among all 35,465 contigs, 37% represented complete chromosomes with repeated C4A4 and T4G4 telomeres (16–96 bp in length with an average size of 38 bp) on both ends (Table 1), while 40% included only one telomere and 23% were assembled without any telomeres (Fig. 1). The average GC content of genomic sequences was found to be 30.71%, which is similar to that observed in other spirotrichs (ranging from 28 to 46%) (Chen et al. 2019; Zheng et al. 2021). No alternative nanochromosome fragmentation was observed in this assembly, consistent with previous observations in other *Euplotes* species (Mozzicafreddo et al. 2021).

**Table 1:** Data for the MAC genome assembly of *Euplotes aediculatus*

Parameter	Two-telomere contigs	One-telomere contigs	Zero-telomere contigs	Total
No. all contigs	13,145	14,105	8215	35,465
No. contigs ≥ 1 kb	11,618	11,772	8215	31,605
No. contigs ≥ 5 kb	1012	1412	1224	3648
No. contigs ≥ 10 kb	105	185	344	634
No. contigs ≥ 25 kb	2	3	47	52
No. contigs ≥ 50 kb	0	0	4	4
Largest contig (bp)	40,119	28,195	70,221	70,221
Smallest contig (bp)	529	500	1000	500
Total length (bp)	33,451,734	37,203,314	26,645,000	97,300,048
*N*_50_ (bp)	3036	3367	4305	3395
*N*_75_ (bp)	2020	2121	2273	2101
*L*_50_ (bp)	3496	3400	1549	8318
*L*_75_ (bp)	6880	6875	3690	17,424
GC (%)	31.09	29.5	31.92	30.71

*N*_50_/*N*_75_, the length of the contig at 50/75% of the cumulative total length of the whole genome. *L*_50_*/L*_75_, the smallest number of contigs whose length sum produces *N*_50_/*N*_75_. No., number of

A total of 43,194 genes were predicted in the genome of *E. aediculatus*. The completeness of gene prediction was assessed by BUSCO, demonstrating its high quality (94.1% complete, 2.3% fragmented, and 3.6% missing genes). Genes were searched against the InterPro protein database using InterProScan, and 31,518 (~ 73.0%) genes were classified with protein functions. Among them, 11,480 genes and 13,349 genes were matched in the Gene Ontology (GO) database and Kyoto Encyclopedia of Genes and Genomes (KEGG) database, respectively.

The predicted genes and their protein-coding sequences (CDS) had average sizes of 1628 bp and 1111 bp, respectively. Introns of *E. aediculatus* were tiny with a peak at 26 bp and exhibited a canonical GT-AG motif (Fig. 1B). Most subtelomeric regions were shorter than 200 bp and the size of 5′ subtelomeric regions (peak at 40 bp) was smaller than that of 3′ subtelomeric regions (peak at 80 bp, Fig. 1C), which was consistent with nanochromosomes reported in other ciliates (Li et al. 2021; Zheng et al. 2022). Analysis of the base composition of the flanking regions within 100 bp from both ends of complete chromosomes revealed two conserved 8-bp motifs (5′-WWATTGAA-3′ and 5′-TTCAATWW-3′) (W = A or T), respectively (Fig. 1D). These motifs were highly reverse complementary and located at similar distances from telomeres (Fig. 1D). One or both motifs were present in 36.4% (4783) of complete chromosomes.

### Genome structure comparison between *Euplotes* species

**Fig. 2:**
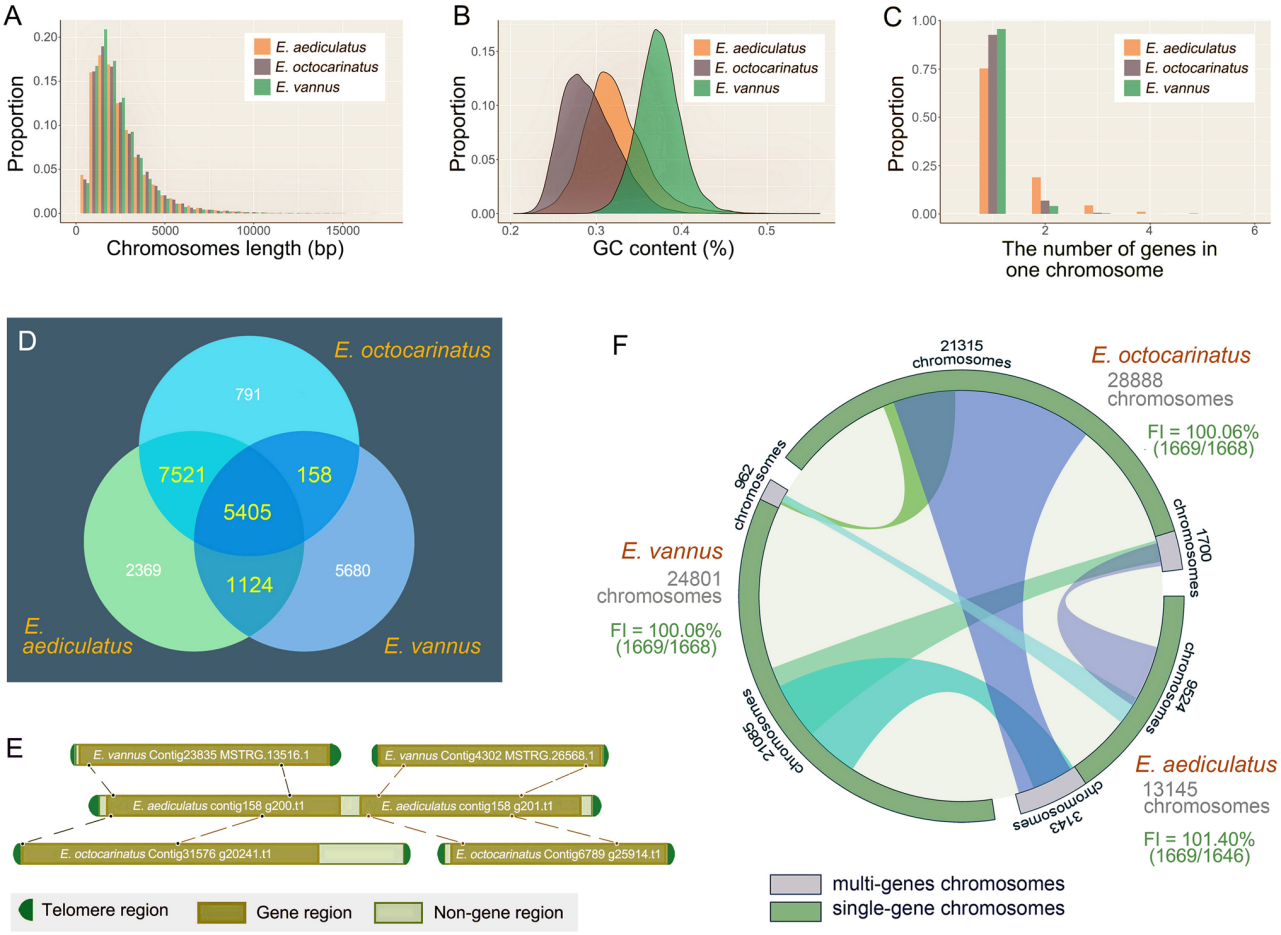
Comparison of genomes among three *Euplotes* species (*E. aediculatus*, *E. octocarinatus*, and *E. vannus*). **A–C** The comparison of chromosome length, GC content, and the number of genes on a single chromosome. Data shown are for complete chromosomes (2-telomere contigs) in the three species. **D** Venn diagram showing the number of overlapping and exclusive orthogroups found in the three *Euplotes* species. **E** A selected example to illustrate homologous genes occupying separate nanochromosomes in one species (*E. vannus* or *E. octocarinatus*), but co-localizing to a multi-gene nanochromosome in another species (*E. aediculatus*). **F** A circle plot showing alignment statistics for homologous genes that occupy separate nanochromosomes in one species, but co-localize to a multi-gene nanochromosome in another species. Corresponding data can be found in Supplementary Table S2. The grey numbers represent the number of complete chromosomes in the existing genome version of the *Euplotes* species. The black numbers represent the number of complete chromosomes with gene annotation in the existing genome version of the *Euplotes* species. Fragmentation Index (FI) = number of homologous genes shared by three *Euplotes* species/number of chromosomes to which they are mapped

A comparison of completed chromosomes (2-telomere contigs) in *E. aediculatus*, *E. vannus,* and *E. octocarinatus* was conducted based on chromosome length, GC content, and number of genes per chromosome (Fig. 2). The distribution of chromosome lengths in all three species were similar, with peaks all fewer than 2500 bp (Fig. 2A). However, the average GC content in *E. vannus* (38%) was higher than that of other two species (31% for *E. aediculatus* and 30% for *E. octocarinatus*) (Fig. 2B), and more *E. aediculatus* chromosomes contained multiple genes (Fig. 2C). The average length of single-gene nanochromosomes in *E. aediculatus* was approximately 2.2 kb, which was similar to that of both *E. octocarinatus* (2.4 kb) and *E. vannus* (2.3 kb).

**Fig. 3:**
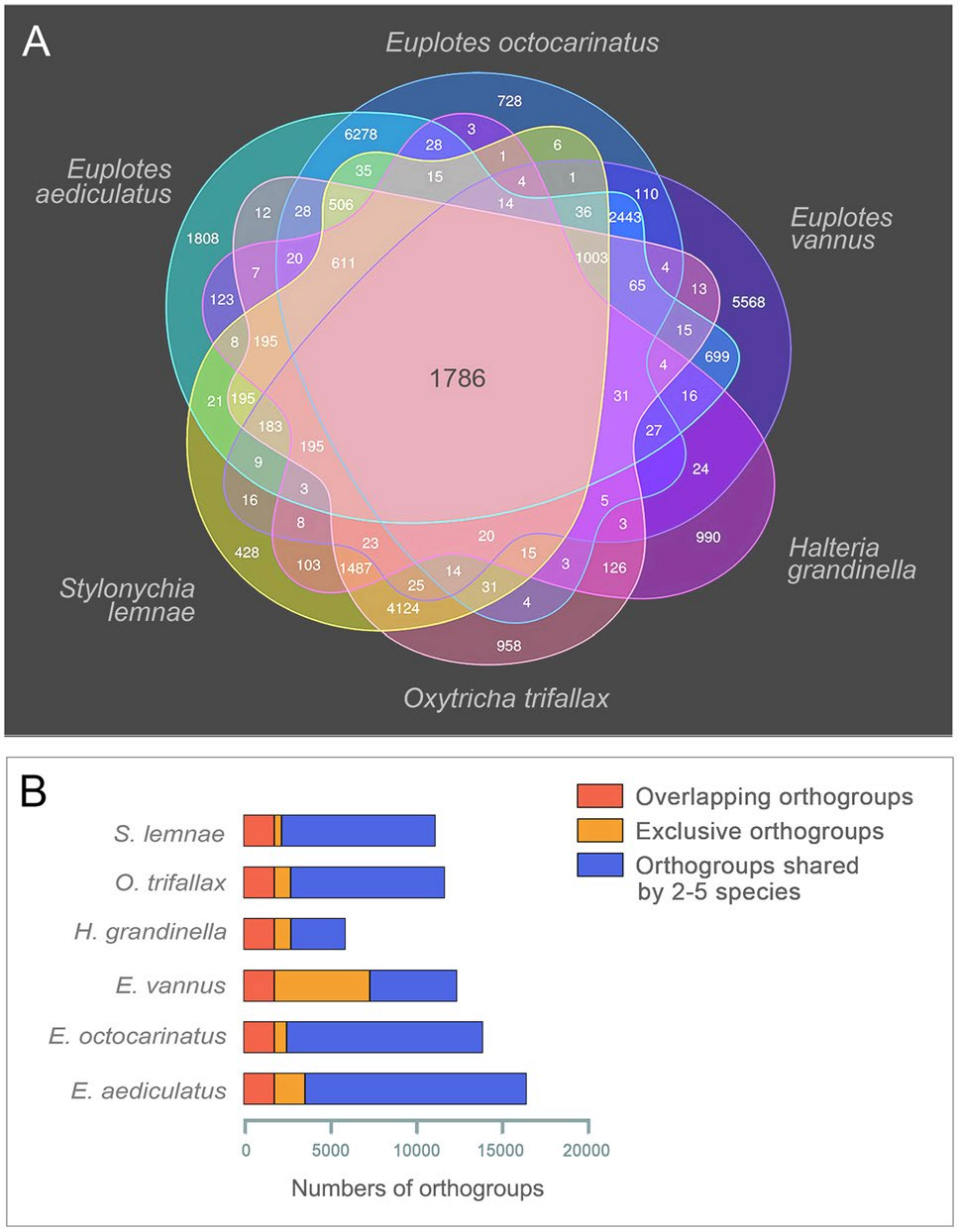
An overview of orthogroups from *Euplotes aediculatus*, *Euplotes vannus*, *Euplotes octocarinatus*, *Stylonychia lemnae*, *Oxytricha trifallax,* and *Halteria grandinella*. **A** A Venn diagram showing the number of overlapping and exclusive orthogroups found in the six species. **B** A bar diagram summarizing orthogroup content in the six species

Orthogroup analyses were performed in the genus *Euplotes* (*E. aediculatus*, *E. vannus* and *E. octocarinatus*) (Fig. 2D) and the class Spirotrichea (*E. aediculatus*, *E. vannus*, *E. octocarinatus*, *Stylonychia lemnae*, *Oxytricha trifallax* and *Halteria grandinella*) (Fig. 3A), respectively. The results showed that the three *Euplotes* species shared 5405 orthogroups while the six spirotrichs shared 1786 orthogroups. Furthermore, *E. aediculatus* shared more orthogroups with *E. octocarinatus* (12,926) than with *E. vannus* (6529), indicating a closer relationship between *E. aediculatus* and *E. octocarinatus*. Among the six spirotrichs mentioned above, *H. grandinella* had significantly fewer orthogroups than the others, while *E. vannus* contained more exclusive orthogroups (Fig. 3B).

An all-vs-all BLASTP search was performed to identify orthologous gene pairs among the three *Euplotes* species. To better understand the structure of complete chromosomes harboring these orthologous genes, genomic sequences between *Euplotes* species were compared (Supplementary Table S2). It was found that some orthologous genes were localized on a nanochromosome exclusively in one species, but co-localized with other genes on a nanochromosome in another species (Fig. 2E, see “1:N” column in Table S2). This reflected the difference in MAC genome fragmentation during *Euplotes* evolution. To quantify this difference, fragmentation index (FI) was defined and calculated as the ratio between the number of homologous genes and the number of chromosomes where they localize (Fig. 2F). The analysis of FI indicated that the MAC genome fragmentation level in *E. aediculatus* was slightly lower than that in the other two *Euplotes* (Fig. 2F).

### Estimation of divergence times and enrichment analysis of expanded gene families

**Fig. 4:**
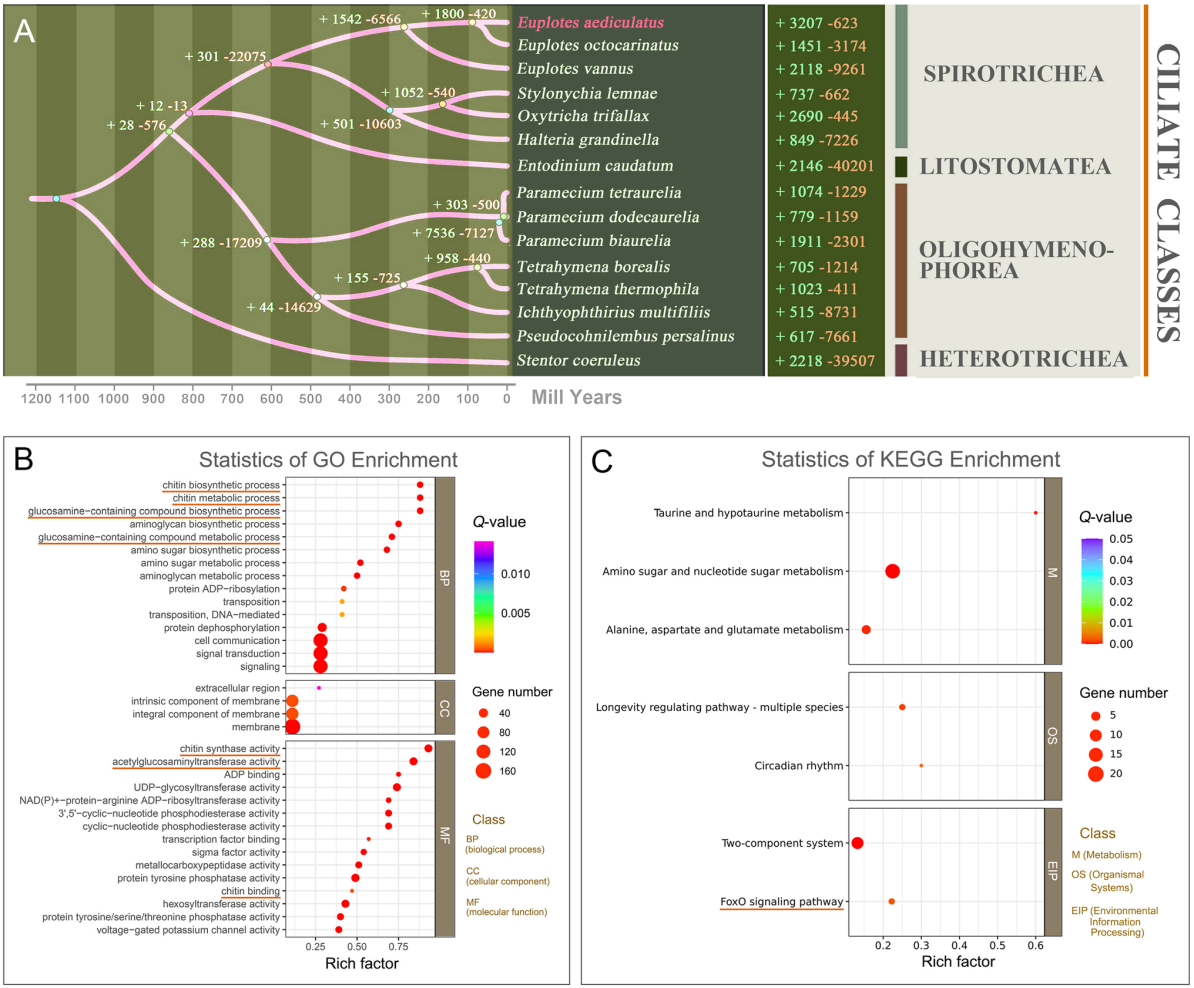
The phylogenetic tree inferred from all amino acid sequences, gene family expansion and contraction, and GO and KEGG pathway enrichment analyses of *Euplotes aediculatus*. **A** The phylogenomic tree, divergence times, and gene family expansion and contraction for *E*. *aediculatus* and 14 other species. The numbers at nodes indicate the number of gene families expanded and contracted at different evolutionary time points. Numbers following species names represent expanded and contracted gene families for that species. **B** GO term enrichment of genes in expansion gene families. Chitin-related terms are underlined in orange. **C** KEGG pathway enrichment of genes in expanded gene families. FoxO signaling pathway is underlined in orange

The speciation times of *Euplotes aediculatus* and 14 other ciliates were inferred based on the phylogenetic analysis of amino acid sequences (Fig. 4A). The *Euplotes* species diverged from other spirotrichs at ~ 610 million years ago (mya). Among euplotids, *E. vannus* was an early branch (~ 265 mya) that experienced gene family contraction. *E. aediculatus* and *E. octocarinatus* diverged from each other at ~ 90 mya, accompanied by a large-scale gene family expansion (3207 gene families in *E. aediculatus*).

GO enrichment analysis was performed for the expanded gene families identified in *E. aediculatus, E. vannus,* and *E. octocarinatus* (Fig. 4B; Supplementary Fig. S1A, C). A total of 152 GO terms were enriched (*p* < 0.05) in *E. aediculatus* (Supplementary Table S4). The top 15 terms after ranking by rich factor values (i.e., the ratio of enriched gene number to all gene number in this pathway term) were collected in each aspect (molecular functions, biological processes, and cellular components). As shown in Fig. 4B*.* *E. aediculatus* exhibited significant enrichment in four chitin-related terms (chitin biosynthetic process, chitin metabolic process, chitin synthase activity, and chitin binding) and three terms related to chitin production, including glucosamine-containing compound biosynthetic process, glucosamine-containing compound metabolic process, and acetylglucosaminyltransferase activity (the monomer of chitin is N-acetylglucosamine). However, chitin-related terms were not found in the enriched GO terms of *E. vannus* and *E. octocarinatus* (Supplementary Fig. S1A, C).

**Fig. 5:**
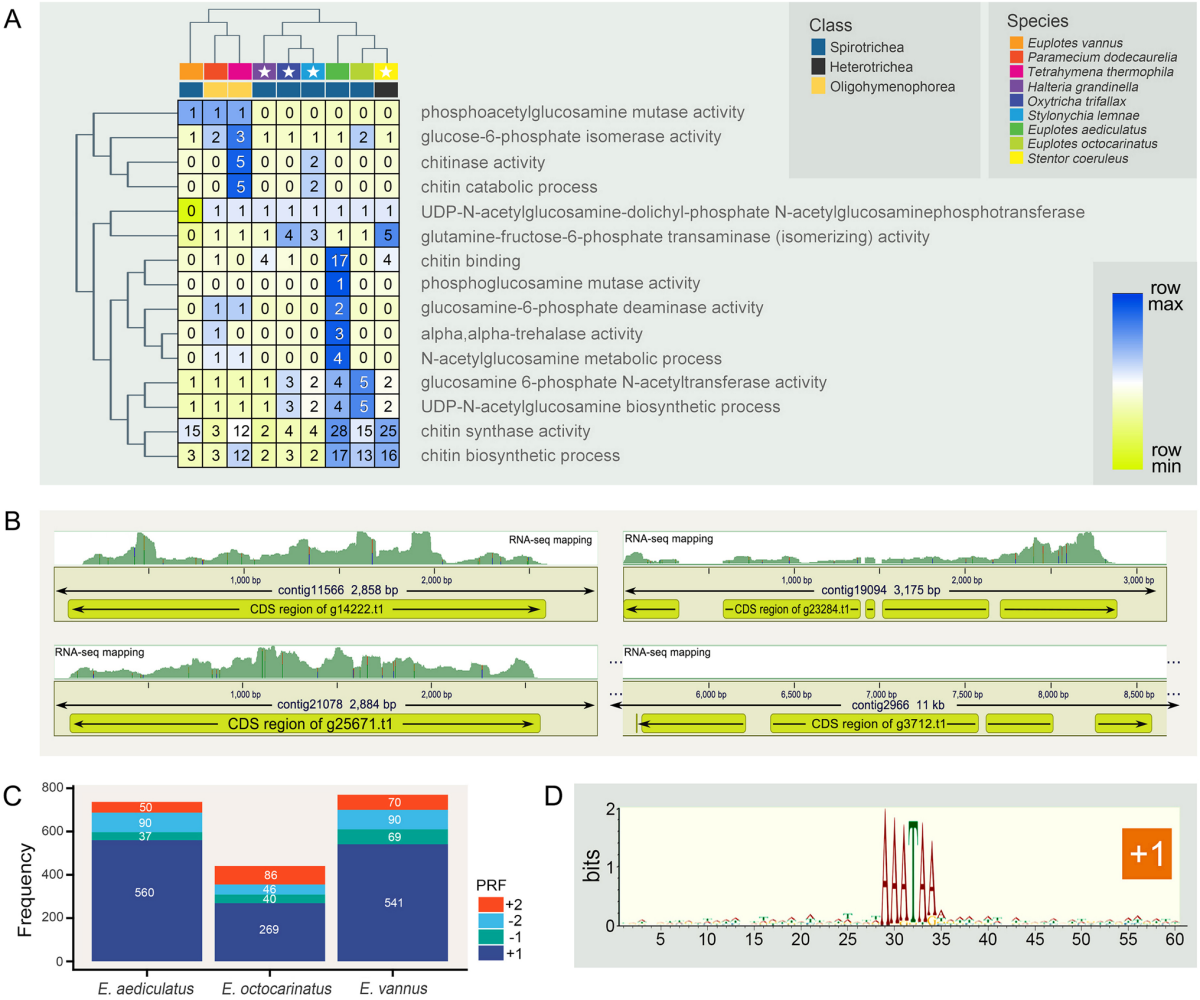
A heatmap of chitin-related GO terms for the nine species, alignment files generated in IGV showing RNA-seq reads mapping to the enriched FoxO signaling pathway related genes and programmed ribosomal frameshifting (PRF) events in three *Euplotes* species. **A** The heatmap of chitin-related GO terms for nine species. For each GO term, the number represents the corresponding number of genes in each species. The white stars indicate that the corresponding species have been reported to produce cysts. Heatmap colors are scaled according to the number of genes in row direction (blue indicates row maximum, yellow indicates row minimum). **B** RNA-seq reads mapped to the four enriched FoxO signaling pathway related genes (g14222.t1, g23284.t1, g25671.t1, g3712.t1) are shown, and g3712.t1 has no RNA-seq mapping. **C** Number of + 1, + 2, − 1 and − 2 PRF events detected in *E. aediculatus*, *E. vannus,* and *E*. *octocarinatus*. **D** The conserved sequence motif associated with + 1 frameshift sites. This analysis is based on the alignment of 30 bp upstream and downstream of the motif from predicted frameshifting events that involve the stop codons TAA or TAG

Chitin-related terms were further selected according to Yang and Fukamizo (2019), and the number of genes associated with these GO terms was checked in nine representatives from the classes Spirotrichea, Heterotrichea, and Oligohymenophorea (Fig. 5A). A total of three enzymes related to chitin synthesis—chitin synthase, glucosamine 6-phosphate N-acetyltransferase, and glucose-6-phosphate isomerase—were shared by all nine ciliates. *E. aediculatus* shared more chitin synthesis-associated genes with *E. octocarinatus* than with *E. vannus*.

To better understand the biological functions of these expanded gene families, the KEGG pathway enrichment analysis was also performed (Fig. 4C). And, the forkhead box O (FoxO) signaling pathway-associated genes were significantly expanded in *E. aediculatus*. The transcriptome data showed that three out of four FoxO signaling genes were expressed (Fig. 5B).

### Programmed ribosomal frameshifting in *Euplotes aediculatus*

In this study, we identified the programmed ribosomal frameshift (PRF) sites in *E. aediculatus* and compared them with that of *E. vannus* and *E. octocarinatus*. The frequencies of four types of frameshifting events (+ 1, − 1, + 2, − 2) are presented in Fig. 5C. A total of 737 frameshifting events (1.7% of all transcripts) were detected in *E. aediculatus*. Among them, + 1 PRF was the most common type and was signaled by a motif of 5′-AAATAR-3′ (R = A or G) (Fig. 5D). No significantly enriched motifs were observed for the other three types of frameshifting events. The proportions of the four types of PRF were similar among the three *Euplotes* species; however, *E. octocarinatus* had approximately half the number of + 1 PRF events compared to *E. aediculatus* and *E. vannus*.

**Fig. 6:**
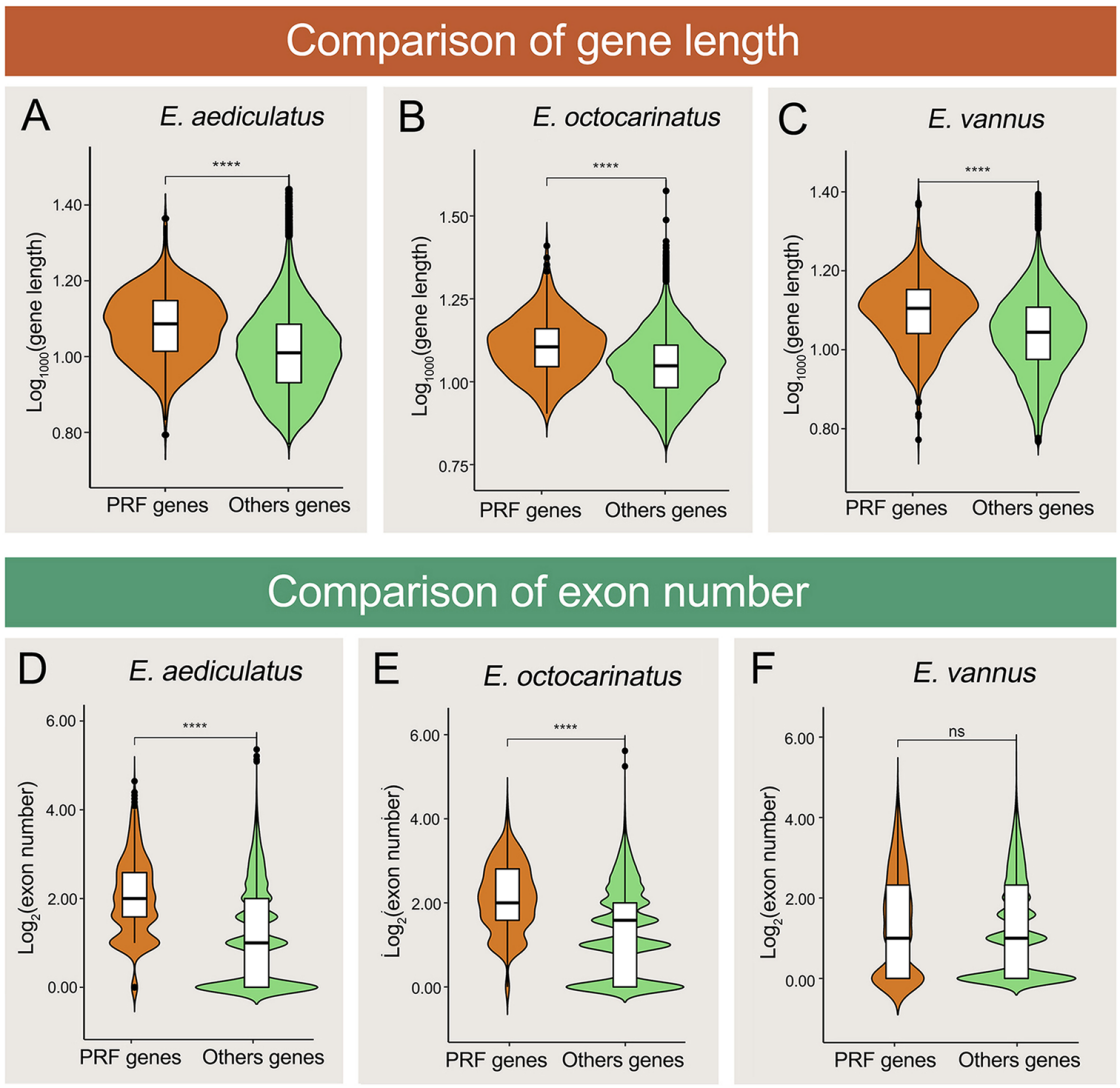
Comparisons of gene length and number of corresponding exons between putative PRF genes and other genes in three *Euplotes* species. **A**–**F** Violin plots showing the difference in gene length (**A**–**C**) and number of corresponding exons (**D**–**F**) between putative PRF genes and other genes

**Fig. 7:**
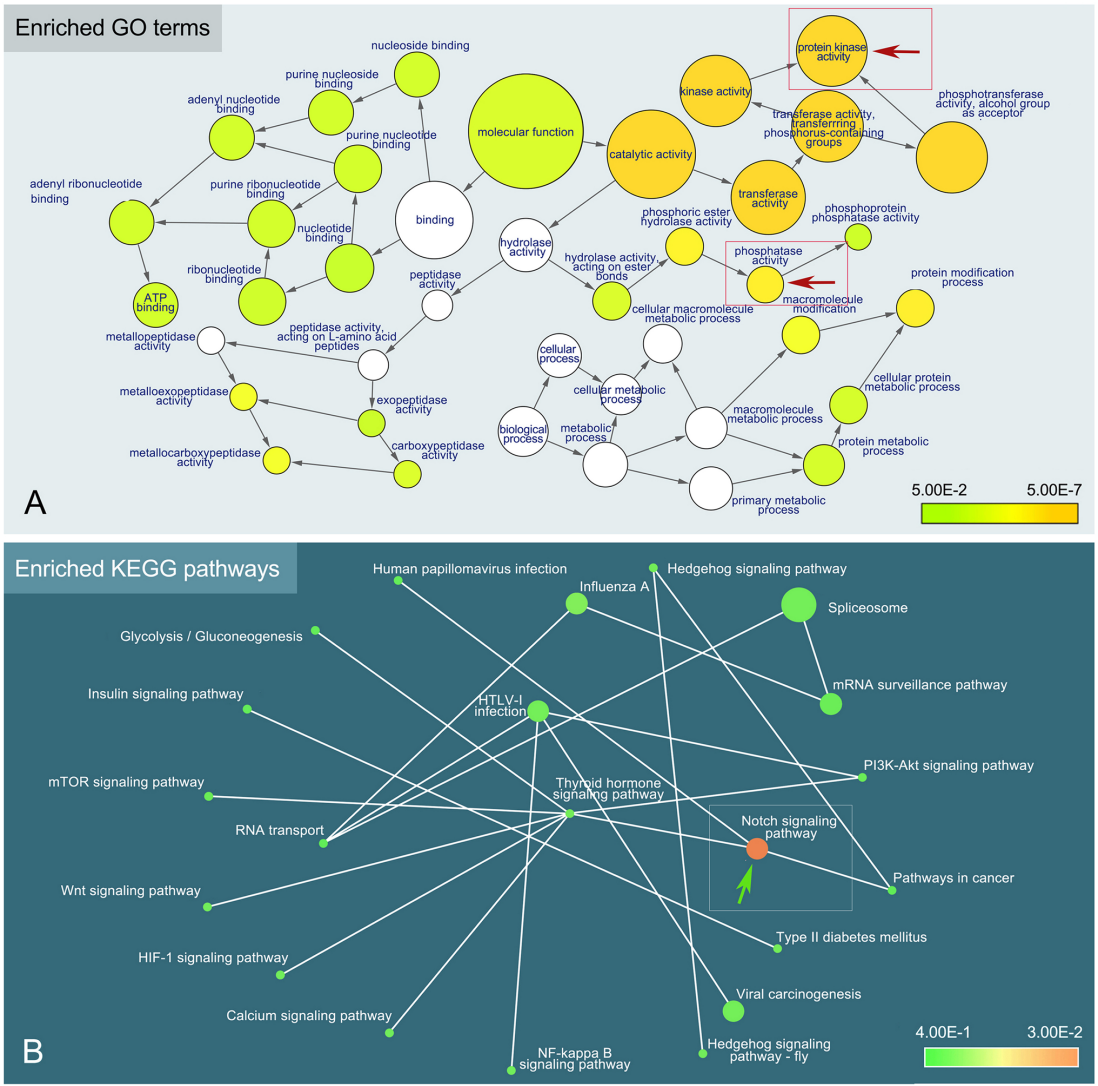
GO term and KEGG pathway enrichment analyses of putative PRF genes in *Euplotes aediculatus*. **A** Enriched GO terms of putative PRF genes analyzed with Bingo. Each GO term is represented by a circle, grey arrows indicate pairs of GO terms with a parent–child relationship. The filled colored circles (nodes) indicate GO terms that are significantly overrepresented (corrected *P* value < 0.05); the deeper of the color, the smaller of the corrected *P* value). Protein kinase and phosphatase activity are marked with red arrows. **B** Enriched KEGG pathways of putative PRF genes analyzed with the OmicShare tools. Each KEGG pathway is represented by a circle. The color gradient color in the circle represents the *Q*-value of KEGG enrichment analysis. White lines indicate that there is a connection between pathways. The size of the circle represents the number of genes enriched in the corresponding pathway

To further investigate the biological significance of PRF, we compared the gene lengths and exon numbers between PRF-associated genes and other genes of the three *Euplotes* species (Fig. 6). Our results revealed that frameshifting events were significantly more frequent in longer genes with more exons (except for *E. vannus*) (*t* test: *P* < 2.22e−16 for Fig. 6A–E, *P* = 0.17 for Fig. 6F). GO enrichment analysis performed on all PRF genes in *E*. *aediculatus* indicated that protein kinases and phosphatases were significantly enriched (*P* value < 0.05) (Fig. 7A). A KEGG pathway enrichment analysis performed on the same set of genes showed that conserved components of the notch signaling pathway were also significantly enriched (*P* value < 0.05) (Fig. 7B). Given that frameshifting events tended to occur in longer genes/proteins, we sought to determine whether PRF proteins were longer than other proteins with identical GO terms or KEGG pathways by comparing their lengths directly with one another’s lengths. Our results indicated that the average lengths of PRF proteins were not consistently longer than that of other proteins with identical GO terms or KEGG pathways (Supplementary Fig. S2), suggesting that the differences in gene/protein length did not contribute to the enrichment observed.

### Evolutionary relationships of euplotids by phylogenomic analyses

**Fig. 8:**
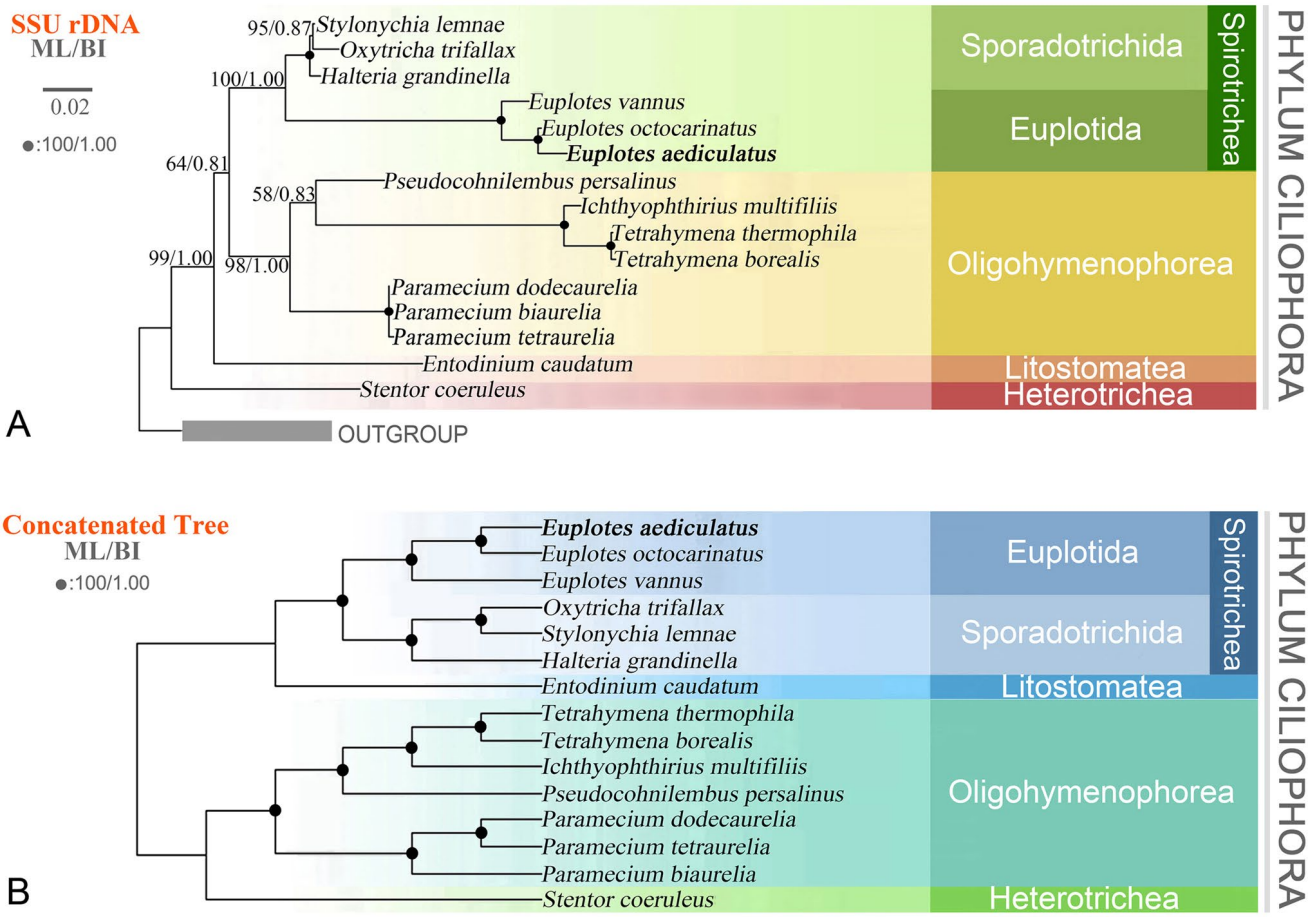
Phylogenetic trees generated from maximum likelihood (ML) and Bayesian inference (BI).** A** A tree inferred from SSU rDNA sequences. **B** A tree based on a concatenation of orthologous protein sequences. Numbers near nodes represent bootstrap values of ML and posterior probabilities of BI. Fully supported (100/1.00) nodes are marked with solid circles. The scale bar corresponds to two substitutions per 100 nucleotide sites

We performed phylogenetic analyses based on SSU rDNA and a concatenated data matrix (comprising 105,832 amino acid sites from 215 orthologous proteins) of 15 representative ciliates using both maximum likelihood (ML) and Bayesian inference (BI) approaches (Fig. 8). The phylogenetic relationships among ciliates were generally consistent between ML and BI trees, as well as between analyses based on SSU rDNA and orthologous protein sequences. *E. vannus* was an early branching clade diverged from *E. aediculatus* and *E. octocarinatus* in both trees.

The systematic position of *Entodinium caudatum* was uncertain. In the SSU rDNA tree, it fell outside of the Spirotrichea + Oligohymenophorea clade with high support (ML/BI, 99%/1.00), but it clustered with Spirotrichea in the concatenated protein tree (Fig. 8). Additionally, *Paramecium dodecaurelia* clustered with *P. tetraurelia*, forming a sister clade to *P. biaurelia* in the concatenated protein tree. However, according to SSU rDNA data they were sister to each other (Fig. 8).

## Discussion

### Overview of the MAC genome of *E. aediculatus*

Our results demonstrate that the MAC genome of *E. aediculatus* is extensively fragmented. In total, 13,145 of the 35,465 contigs generated in this study are assembled into completed 2-telomere nanochromosomes, with 9500 of these encoding only one gene (Fig. 2C). Most introns are tiny and highly conserved on both sides of the sequence, with a peak length of 26 bp, though many are longer and push the overall mean length to 199 bp (Fig. 1B). This finding is consistent with that of other reported spirotrichous ciliates (Chen et al. 2019; Swart et al. 2013; Zheng et al. 2021, 2022).

Chromosome fragmentation in the zygotic nucleus typically occurs at or near the chromosome breaking sites (CBSs), after which fragmented chromosomes undergo further processing and are coupled with de novo telomere to form complete macronuclear chromosomes (Chang et al. 2004; Duan et al. 2021; Jahn and Klobutcher 2002; Swart et al. 2013). In *Euplotes* species, CBSs are duplicated and retained in the MAC genome during conjugation (Chen et al. 2019; Jahn and Klobutcher 2002). The motifs we identified in subtelomeric regions (Fig. 1D) resemble motifs (5′-TTGAA-3′ and 5′-TTCAA-3′) previously reported in CBSs of *E. vannus* and *E. crassus* (Chen et al. 2019; Jahn and Klobutcher 2002). Furthermore, the positional distribution of our two motifs within subtelomeric region (Fig. 1D) is consistent with that observed for *E. vannus* and *E. crassus* (Chen et al. 2019; Jahn and Klobutcher 2002), suggesting that these motifs may play a role in the chromosome fragmentation at CBSs during macronuclear chromosomal rearrangements.

### Nanochromosomes in spirotrichous ciliates

Previous studies of spirotrichous ciliates have reported highly fragmented MAC genomes comprising 12,000 to 29,000 nanochromosomes (Chen et al. 2019; Li et al. 2021; Swart et al. 2013; Zheng et al. 2021), which is in accordance with our findings for *E. aediculatus* (Fig. 2). In general, the nanochromosomes of spirotrichs exhibit several common characteristics. First, they are typically short in length, averaging only 1–3.5 kb (Li et al. 2021). As an example, the shortest complete nanochromosome in *Halteria grandinella* is only 345 bp (Zheng et al. 2021). In our study, *E. aediculatus* has a similar average nanochromosome length (about 2.5 kb; minimum 529 bp). Second, most nanochromosomes contain only one gene and their lengths appears to be determined by the length of their respective genes. The number of genes located on multi-gene nanochromosomes is small; for instance, the maximum number of genes on a single chromosome is up to eight (*E. aediculatus*), four (*E. octocarinatus*) and five (*E. vannus*). Third, structural sequences within the nanochromosomes—including telomeres subtelomeric regions and introns—are shortened during evolution (Chen et al. 2019; Li et al. 2021; Swart et al. 2013; Zheng et al. 2021). Consistent with the small size of nanochromosomes, most introns and subtelomeric non-genic regions in *E*. *aediculatus* are less than 50 bp and 200 bp in size, respectively (Fig. 1B, C), and the average telomere length is only 38 bp.

Previous study demonstrated that the removal of CBS eliminates chromosome fragmentation, and moving CBS to a new location creates a new site for chromosomal fragmentation (Jahn and Klobutcher 2002). Chang et al. (2004) proposed that mutations or deletions in CBSs result in the failure of genome fragmentation during macronuclear development, leading to chromosomes with multiple genes. By comparing the locations of orthologous gene pairs (Fig. 2E, F; Supplementary Table S2), the fragmentation index (Fig. 2F), and the proportion of single-gene chromosomes (Fig. 2C; Supplementary Table S3), we find that *E. octocarinatus* and *E. vannus* have slightly higher level of genome fragmentation than *E. aediculatus*. Each euplotid species has its species-specific multi-gene nanochromosomes (see “Misaligned N” column in Supplementary Table S2), indicating that: (1) CBS loss occurs in all three euplotids; (2) CBS loss is not gene-specific—chromosomes and genes involved vary among species. Among the three euplotids, CBS loss is most pronounced in *E. aediculatus*. Additionally, differences in fragmentation level of MAC genome within the class Spirotrichea (Supplementary Fig. S3, Table S3) may be due to the varying degrees of CBS loss among different species. As mentioned previously, the MAC genome of ciliates is formed from a zygotic nucleus through a series of genome-wide rearrangements. Thus, we extract the intergenic region sequences from all multi-gene nanochromosomes in the three euplotid species and find no evidence of CBS motifs within these regions. This finding may indicate that CBSs have evolved within the MIC genomes. We anticipate that additional evidence regarding these genome evolution events will be uncovered through further study of other ciliates with nanochromosomes.

### Chitin synthesis and FoxO signaling pathway

Chitin can protect organisms against environmental mechanical or chemical stress, and can act as a structural component that contributes to cell shape (Mulisch 1993; Mulisch and Hausmann 1989). In insects, the chitin biosynthetic pathway involves several enzymes including trehalase, glucose-6-phosphate isomerase, glutamine-fructose-6-phosphate aminotransferase, glucosamine-6-phosphate N-acetyltransferase, phosphoglucosamine mutase, and chitin synthase (CHS) (Yang and Fukamizo 2019). CHS is highly conserved enzyme and the presence of CHS gene in the genomes of organisms can be considered diagnostic for the capability of chitin biosynthesis (Yang and Fukamizo 2019). A heatmap of above chitin-related GO terms for nine representative ciliates shows conserved orthologs within the phylum (Fig. 5A). Given that (1) previous studies have detected chitin in lorica (a shell-like protective outer covering of ciliates) and cyst walls of various ciliates (including other *Euplotes*), and (2) CHS gene is detected in all nine species (Fig. 5A), our results provide further evidence that chitin synthesis is an ancestral characteristic of ciliates (Greco et al. 1990; Yang and Fukamizo 2019). However, since five of the nine ciliates included in our study have not been reported to form cysts, it is not possible to predict the function of chitin in these ciliates.

*Euplotes aediculatus* exhibits the greatest expansion of gene families among the 15 ciliate species in this study (Fig. 4A). Interestingly, the top five GO terms with the highest enrichment factor among expanded gene families are related to chitin (Fig. 4B). We speculate that the expansion of chitin-related gene families in *E. aediculatus* may be due to environmental stresses—a harder cyst wall would increase its chance of survival in harsh environment. The specific environmental conditions that led to the expansion of chitin synthesis genes in *E. aediculatus* are unknown, and will require further investigation into the challenges faced by this species. In addition, *E. octocarinatus* has more chitin-related gene family members than *E. vannus* (Fig. 5A). Given that the marine environment inhabited by *E*. *vannus* is more stable than the freshwater environment where *E. aediculatus* and *E*. *octocarinatus* occur (Herlemann et al. 2011; Woodward et al. 2010), this finding may suggest that the variable habitat of the latter two species has a stronger selection effect on chitin-related genes than that of *E*. *vannus*.

KEGG annotation shows that the FoxO signaling pathway is also enriched among expanded gene families in *E. aediculatus* (Fig. 4C). This pathway regulates gene expression in many cellular physiological events, including apoptosis, cell-cycle control, glucose metabolism, oxidative stress resistance, and longevity (Carter and Brunet 2007). Several studies focusing on invertebrates and mammals have shown that transcription factors within the FoxO family improve stress resistance and longevity when activated (Ikeda et al. 2017; Kahn 2014; Seo et al. 2015). Similarly, the FoxO signaling pathway may play a role in responding to and adapting to adverse environmental stresses that lead to encystment in *Pseudourostyla cristata* (Ciliophora, Spirotrichea, Hypotrichida) (Pan et al. 2019a). Thus, in addition to the expansion of chitin-related gene families, *E. aediculatus* may also use the FoxO signal pathway to increase the survival of cysts. These survival strategies may contribute to the worldwide distribution of *E. aediculatus* (Abraham et al. 2021; Curds 1975).

### Programmed ribosomal frameshifting in *E. aediculatus*

Programmed ribosomal frameshifting (PRF) is a recoding event that shifts the ribosomal reading frame at a specific position during translation. PRF sites are prevalent in euplotids and are associated with highly conserved nucleotide sequence motifs (Chen et al. 2019; Wang et al. 2016). All four types of frameshifting events are found in *E. aediculatus* and + 1 frameshifting is predominant among these events with a conservative motif (Fig. 5C, D). However, the numbers of PRF sites in *E. vannus* and *E*. *octocarinatus* are lower than those reported in previous studies (Chen et al. 2019; Wang et al. 2016). This discrepancy may be an artifact of changes to the ciliate library used for putative protein alignment and the specific filter conditions, as the protein library used here is newly constructed from the 12 non-*Euplotes* ciliates (Supplementary Table S1), and the FScanR software used to identify PRF is set with more stringent thresholds (E-value cutoff = 1e−10, mismatch_cutoff = 100, frameDist_cutoff = 10).

Protein phosphorylation and dephosphorylation regulate many essential cellular functions (Graves and Krebs 1999). GO enrichment analysis of putative PRF genes in *E. aediculatus* indicates that enzymes responsible for protein phosphorylation (protein kinases) and dephosphorylation (phosphatase) are significantly enriched (Fig. 7A). This finding is consistent with that for *E. octocarinatus*, which showed that putative + 1 PRF genes are significantly enriched in the regulation of various biological processes (Wang et al. 2016). KEGG enrichment analysis of putative PRF genes in *E. aediculatus* reveals that components of the notch signaling pathway are significantly enriched (Fig. 7B). The notch signaling pathway in metazoa is involved in cellular development including proliferation and apoptosis (Wang et al. 2009). Given that no studies have examined this pathway in ciliates, we can only speculate that homologs of notch signaling pathway genes in *E. aediculatus* might play a similar role in cellular development. In summary, our results support the observation that PRF genes play important roles in regulating many cellular events in *Euplotes*.

### Phylogenetic analyses

Our phylogenetic trees constructed using SSU rDNA (Fig. 8A), orthologous protein amino acid (Fig. 8B), and all protein amino acid sequences (Fig. 4A) all indicate similar topologies for the clade of *Euplotes*. These findings correspond to the GC content result where *E. aediculatus* has a similar average GC content to *E. octocarinatus* but lower than *E. vannus* (Fig. 2B). *Entodinium caudatum* (class Litostomatea) has a close relationship with the Spirotrichea in two concatenated protein trees (Figs. 4A, 8A), consistent with studies based on concatenated data (Gao et al. 2016; Pan et al. 2019b; Wang et al. 2021). In addition, the two concatenated protein trees provide a clearer guide to the evolutionary relationships among *Paramecium* spp. compared with the SSU rDNA trees (Figs. 4A, 8). Hence, our study further supports the view that concatenate data might possess higher resolution than single SSU rDNA barcoding marker (Gao et al. 2016; Pan et al. 2019b).

## Conclusion

We report the MAC genome assembly of a representative euplotid species, *Euplotes aediculatus*. This highly fragmented genome consists of a large number of nanochromosomes, just like other MAC genomes in spirotrichs but with a different fragmentation level. Differences in fragmentation levels of MAC genomes among various spirotrichs may arise from the different degrees of CBS loss. In addition, the gene functional analysis suggests that the gene families expanded in *E. aediculatus* are associated with chitin metabolism and FoxO signaling pathway, which could be relevant for improving the survival of cysts. All nine representative ciliates have the capability for chitin biosynthesis while only four of them are reported with cysts. So, the function of chitin in other five ciliates require further investigation. PRF tends to occur in longer genes with more exons and related genes play important roles in regulating many cellular processes in *Euplotes*. Comparative genomics analyses show that *Euplotes* species exhibit a relatively high diversity of genomic features in the genomic GC content, genome fragmentation level, expanding gene families, and the frequency of PRF events, which may associate with the respective direction of adaptive evolution for each species. Multi-angle genomics analyses indicate that *E. aediculatus* and *E. octocarinatus* have a closer phylogenetic relationship. These results provide new insights into the evolution of nanochromosomes and gene function, and contribute to our growing understanding of the diversity of MAC genomic features in *Euplotes* and, to a larger extent, ciliates.

## Materials and methods

### Cell culture, DNA and RNA extraction, and Illumina sequencing

*Euplotes aediculatus* was collected from a freshwater pond in Zhongshan Park (36° 4′ 14.28″ N, 120° 21′ 26.51″ E), Qingdao, China. The species identification relied on morphological features in vivo and silver staining (Curds 1975), as well as the nuclear small subunit ribosomal DNA (SSU rDNA) sequence (Sogin et al. 1986). Ten cells were isolated and cultured at 25 °C using double distilled water with *Escherichia coli* as food, until the ciliates reached about 10^6^ cells/L. After cells were harvested by centrifugation for 3 min at 200 *g*, the genomic DNA was extracted using the DNeasy Blood & Tissue kit (QIAGEN, #69504, Germany) according to the manufacturer's instructions. Total RNA was extracted using the RNeasy Plus mini kit (QIAGEN, #74134, Germany).

Two DNA libraries and one RNA library were constructed with NEBNext Ultra DNA Library Prep Kit for Illumina (NEB, #E7370L, USA) and NEBNext Ultra RNA library prep kit for Illumina (NEB, #E7530S, USA), respectively. The paired-end sequencing with a read length of 150 bp was performed on the Illumina Hiseq2500 platform. Finally, the sequencing adapters were trimmed, and low-quality reads were filtered out using Fastp (-q 20 -u 40 -l 36) (Chen et al. 2018).

### Genome assembly

After filtering of the low-quality and duplicate reads, approximately 53.59 Gb of 150 bp paired-end (PE150) data were retained for assembly. The MAC genome was de novo assembled using MEGAHIT version 1.2.9 (Li et al. 2015) with the default parameters, producing the primary contigs. CD-HIT software (CD-HIT-EST) (Li and Godzik 2006) was used with a threshold of 0.95 to identify possible alternative nanochromosome fragmentation in the primary contigs (Mozzicafreddo et al. 2021). Then the primary contigs were merged with Cap3 (version date: 02/10/15) (Huang and Madan 1999). Contaminant contigs of mitochondria and bacteria were identified by searching against mitochondrial genomes of ciliates and genome sequences of bacteria downloaded from GenBank (Benson et al. 2012) using BLASTN (v2.10.1 +) (E-value cutoff = 1e−5). In addition, low-quality contigs (GC > 50% or coverage < 5 × contigs) were excluded.

Redundans (-identity 0.55 -overlap 0.8) (Pryszcz and Gabaldón 2016) was employed to eliminate the redundancy of the remaining contigs, obtaining the final assembly result. The quality of the genome assembly was evaluated using QUAST version 5.0.2 (Gurevich et al. 2013).

### Gene prediction and annotation

RNA-seq reads of *E*. *aediculatus* were mapped to the MAC genome assembly by HISAT2 version 2.1.0 (Kim et al. 2015), and the transcripts were assembled by StringTie version 1.3.7 (Pertea et al. 2016). The assembled transcriptome was analyzed with TransDecoder version 1.3 (https://github.com/TransDecoder/TransDecoder) (*Euplotes* genetic codes) to predict candidate coding regions. RepeatMasker version 4.0.7 (Smit 1996) was employed to mask repetitive nucleotide sequences in the MAC genome. The de novo gene prediction was performed using AUGUSTUS version 3.4.0 (Keller et al. 2011) with hints of RNAseq data. BUSCO version 4.1 (Simão et al. 2015) was used to evaluate the completeness of the predicted gene set with Alveolata databases. The sequences of introns were extracted using the TBtools software (Chen et al. 2020a). The motifs in extracted sequences were searched using MEME (version 5.5.0), using default parameters (Bailey et al. 2006).

Protein domains and other functional elements were detected and annotated using InterProScan version 5.52–86.0 (Jones et al. 2014). Gene Ontology (GO) (Gene Ontology Consortium 2015) and Kyoto Encyclopedia of Genes and Genomes (KEGG) (Kanehisa et al. 2012) annotations for each gene were extracted from the corresponding InterProScan and KofamScan (Aramaki et al. 2020) results.

### The identification of orthologous gene pairs and the calculation of fragmentation index (FI)

Protein sequences were compared to identify orthologous gene pairs among three *Euplotes* species (*E. aediculatus*, *E. octocarinatus* and *E. vannus*) using all-vs-all BLASTP search (with alignment length ≥ 50, E-value ≤ 1e−5). The number of homologous genes shared by three *Euplotes* species and the number of chromosomes related to the homologous genes were used to calculate the FI value (FI = number of homologous genes shared by three *Euplotes* species/number of chromosomes to which they are mapped). About the calculation of the gene number of each chromosome, we kept only the longest isoform for downstream analysis.

### Gene family analysis and divergence time estimation

The genome and amino acid sequences of 14 other ciliates were collected from different sources (Supplementary Table S1), to perform the gene family and phylogenetic analyses. All amino acid sequences of *E*. *aediculatus* were combined with those of the 14 additional taxa and employed to identify orthologous gene clusters using OrthoFinder version 2.5.4 (-S diamond -M msa -T raxml) (Emms and Kelly 2015). A rooted species tree (SpeciesTree_rooted.txt inferred by OrthoFinder) was used to produce an ultrametric phylogenetic tree by the program r8s (Sanderson 2003). This ultrametric phylogenetic tree was applied by CAFE version 4.2.1 (Computational Analysis of gene Family Evolution) to identify rapidly expanded/contracted gene families with a *P* value cutoff of 0.01 (Han et al. 2013). For the divergence time analysis, the divergence time of *Paramecium tetraurelia* and *Tetrahymena thermophila* (median time: 609.8 MYA) was obtained through the TimeTree (http://timetree.org/) as the reference. Subsequently, they were used as an input parameter for r8s to estimate the divergence time of all 15 ciliates.

### Enrichment analysis and detection of programmed ribosomal frameshifting

The GO term and KEGG pathway enrichment analyses of significantly expanded gene families were performed using the R package, clusterProfiler (Yu et al. 2012).

Programmed ribosomal frameshifting was detected by aligning transcript sequences against the corresponding protein library using BLASTX (E-value cutoff = 1e−5) (Altschul et al. 1990; Chen et al. 2019). The hit result was analyzed by R package FScanR (E-value cutoff = 1e−10, frameDist_cutoff = 10 nt, mismatch_cutoff = 100) (https://github.com/seanchen607/FScanR) to obtain the results of PRF events (Chen et al. 2020b).

### Phylogenetic analysis

For the SSU rDNA tree, the SSU rDNA sequence of *Euplotes aediculatus* (derived from the assembled genome) was aligned with those of 14 other ciliates on the GUIDANCE web server (Penn et al. 2010) with default parameters. Both ends of the alignment were trimmed manually using Bioedit version 7.2.6 (Hall 1999). A maximum likelihood (ML) tree was produced with RAxML-HPC2 on XSEDE v.8.2.12 in the CIPRES Science Gateway (Miller et al. 2010). The reliability of internal branches was assessed using a nonparametric bootstrap method with 1000 replicates. Bayesian inference (BI) analysis was performed on CIPRES Science Gateway by MrBayes on XSEDE v.3.2.6 (Ronquist et al. 2012) using the GTR + I + G model (selected by MrModelTest v.2.2). Markov chain Monte Carlo simulations were run with two sets of four chains for 2 × 10^6^ generations with a sample frequency of 100 generations. The first 5000 trees (25%) were discarded as burn-in. All remaining trees were used to calculate posterior probabilities using a 50% majority rule consensus. Phylogenetic trees were visualized via MEGA version 5.0 (Tamura et al. 2011) and SeaView v.4 (Gouy et al. 2010).

The orthologous proteins alignment of the 15 ciliates species generated by Orthofinder was used to perform the phylogenomic analysis. The ML tree was generated using IQ-TREE version 2.1.4 (-m MFP -B 1000 -bnni) (Nguyen et al. 2015). The BI analysis was performed using PhyloBayes version 4.1c (-cat –gtr –x 5 10,000) (Lartillot et al. 2013).

## Supplementary Information

Below is the link to the electronic supplementary material.

Supplementary file1 (PDF 1787 kb)

Supplementary file2 (XLSX 40 kb)

## Data Availability

The final genome assembly, genomic reads, and RNA-seq reads have been deposited in GenBank (genome assembly: JAQGFW000000000, genomic reads: SAMN31782627 and SAMN31782628, RNA-seq reads: SAMN31782629). Genome assembly and gene annotation data including coding regions and predicted protein sequences are also available in https://evan.ciliate.org/.
