# Supplementary material for: Comparative genome analysis of three euplotid protists provides insights into the evolution of nanochromosomes in unicellular eukaryotic organisms
Source: Mar Life Sci Technol. 2023 May 28;5(3):300–15. doi: 10.1007/s42995-023-00175-0 (PMC10449743; doi:10.1007/s42995-023-00175-0)
Supplement: Supplementary file 1 — Supplementary file1 (PDF 1787 kb) [file 42995_2023_175_MOESM1_ESM.pdf]

Comparative genome analysis of three euplotid protists provides insights into the evolution of nanochromosomes in unicellular eukaryotic organisms

Didi Jin[1,#] · Chao Li[2,#] · Xiao Chen[3,#] · Adam Byerly[4] · Naomi A. Stover[5] · Tengteng Zhang[1] · Chen Shao[1,*] · Yurui Wang[1,*]

[1] *Laboratory of Protozoological Biodiversity and Evolution in Wetland, College of Life Sciences, Shaanxi Normal University, Xi'an 710119, China*
[2] *Institute of Evolution & Marine Biodiversity, Ocean University of China, Qingdao 266003, China*
[3] *Laboratory of Marine Protozoan Biodiversity and Evolution, Marine College, Shandong University, Weihai 264209, China*
[4] *Department of Computer Science and Information Systems, Bradley University, Peoria 61625, USA*
[5] *Department of Biology, Bradley University, Peoria 61625, USA*

[#] These authors contributed equally to this work.

***Correspondence:** Chen Shao, email: shaochen@snnu.edu.cn; Yurui Wang, email: wangyurui@snnu.edu.cn

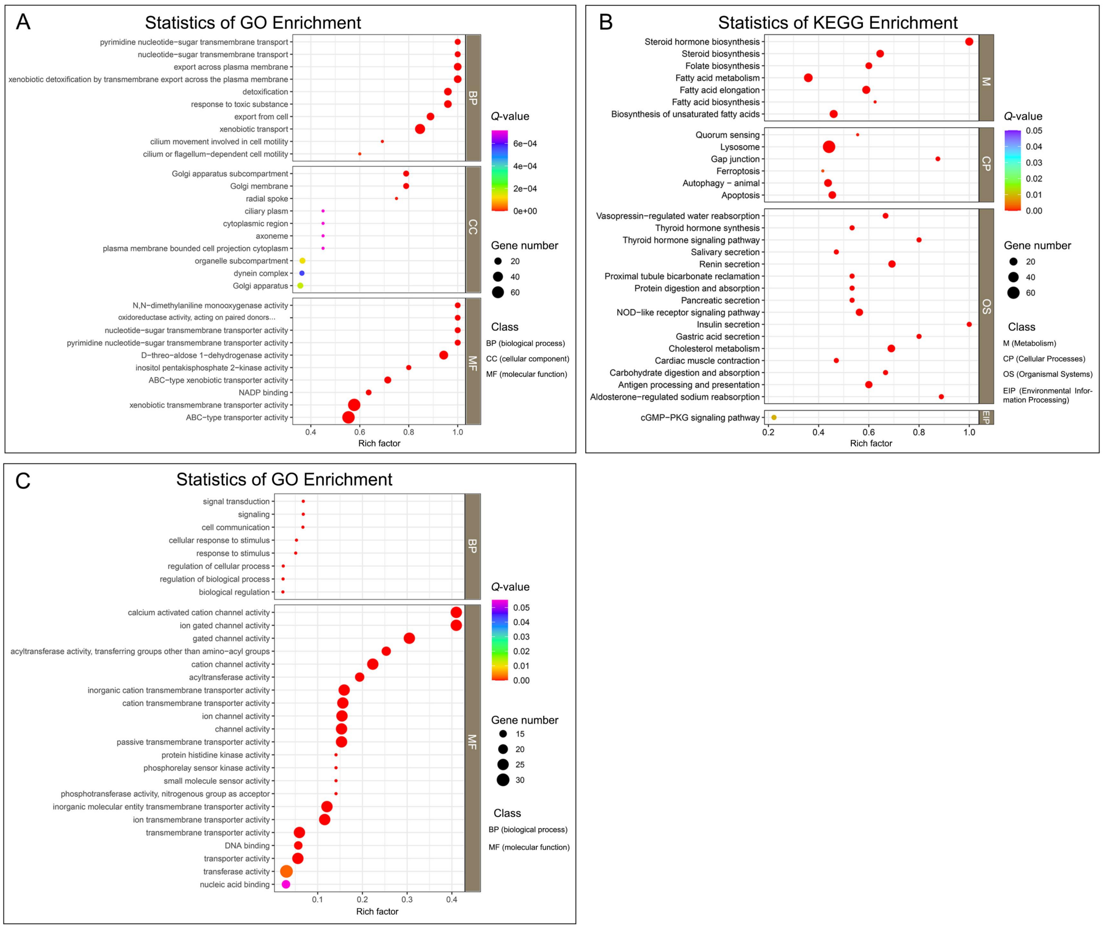

Fig. S1 GO term and KEGG pathway enrichment analysis of *Euplotes octocarinatus*, and GO term enrichment analysis of *Euplotes vannus*. **a** GO term enrichment of genes in expanded gene families for *Euplotes octocarinatus*. **b** KEGG pathway enrichment of genes in expanded gene families for *Euplotes octocarinatus*. *Euplotes vannus* was not enriched for any KEGG pathway. **c** GO term enrichment of genes in expanded gene families for *Euplotes vannus*.

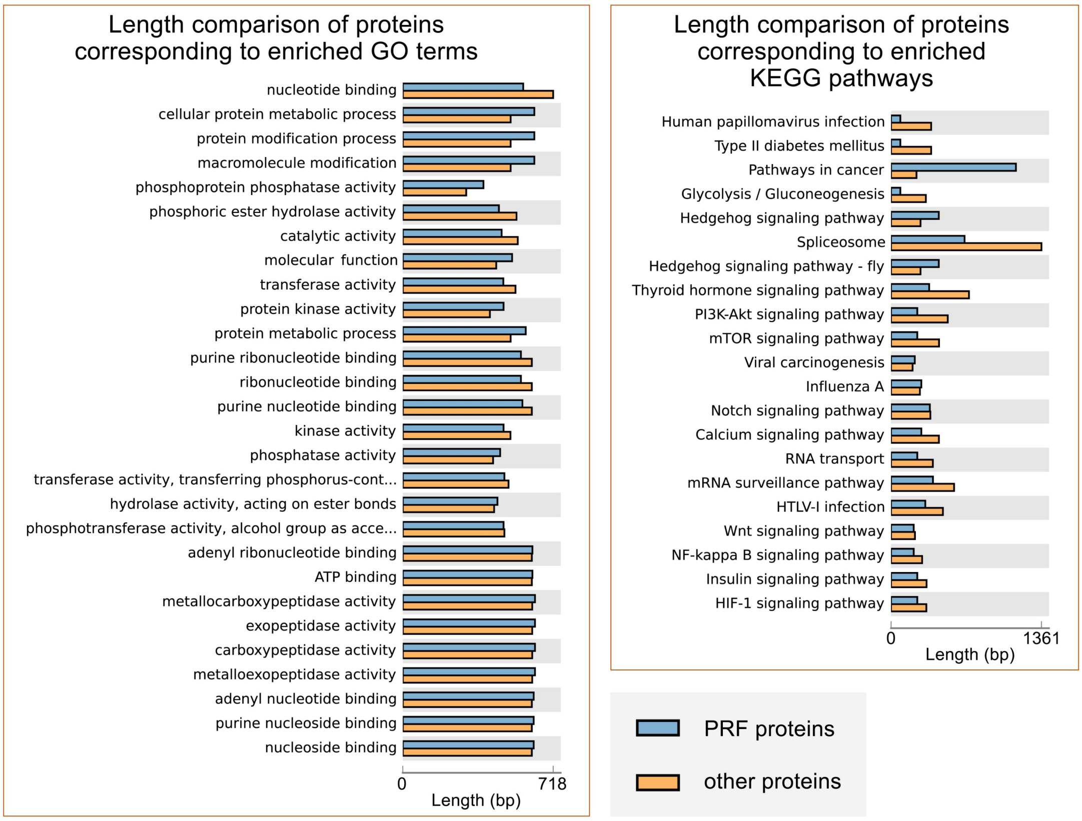

Fig. S2 Length comparison of PRF protein length and other protein length with the same GO terms or KEGG pathways.

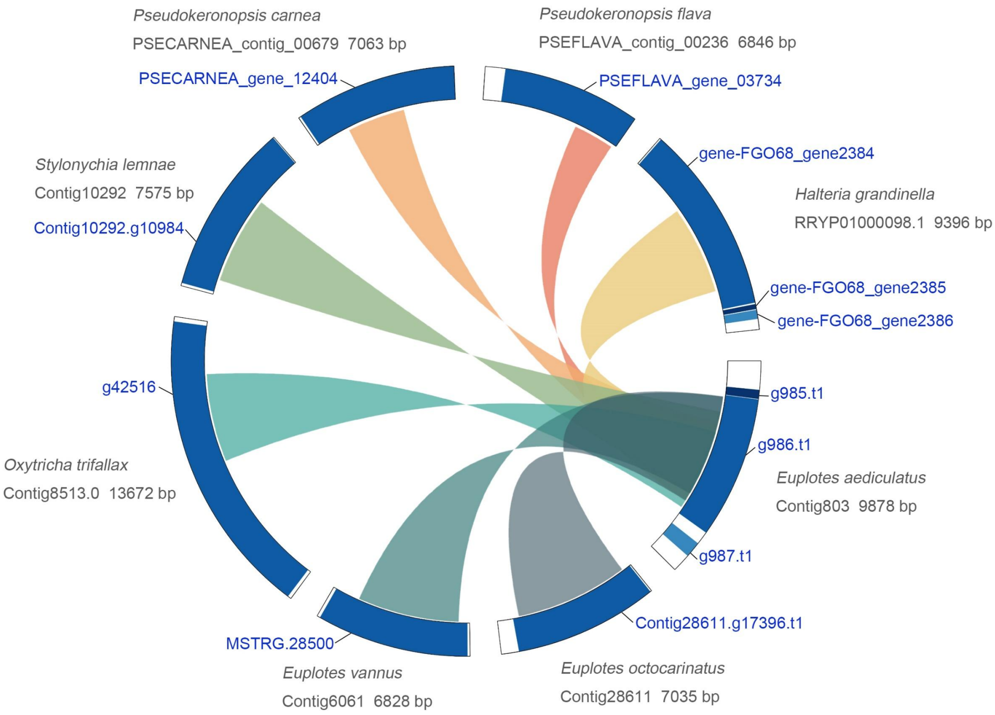

Fig. S3 A selected example for eight spirotrichs to illustrate homologous genes occupying separate nanochromosomes in one species, but co-localizing to a multi-gene nanochromosome in another species, showing the difference in the location of orthologous gene pairs in Spirotrichea. Information about species and chromosome are in grey font, while gene names are in blue font.

Table S1 Description of macronuclear (MAC) genomes and amino acid sequences sources of 15 ciliates

Species	SSU rDNA sequences sources	MAC genomes and amino acid sequences sources	Order	Class
Stentor coeruleus	From NCBI, Accession: JQ282899	http://ciliates.org/	Heterotrichida	Heterotrichea
Entodinium caudatum	From MAC genome	asked data from author	Entodiniomorphida	Litostomatea
Ichthyophthirius multifiliis	From NCBI, Accession: U17354	http://ciliates.org/	Ophryoglenida	Oligohymenophorea
Pseudocohnilembus persalinus	From NCBI, Accession: AY835669	http://ciliates.ihb.ac.cn/	Philasterida	
Paramecium tetraurelia	From NCBI, Accession: KY852452	https://www.ncbi.nlm.nih.gov/	Peniculida	
Paramecium dodecaurelia	From MAC genome	https://www.ncbi.nlm.nih.gov/		
Paramecium biaurelia	From NCBI, Accession: MG009438	https://www.ncbi.nlm.nih.gov/		
Tetrahymena thermophila	From NCBI, Accession: X56165	http://ciliates.org/	Tetrahymenida	
Tetrahymena borealis	From NCBI, Accession: MH051920	http://ciliates.ihb.ac.cn/tcgd/		
Stylonychia lemnae	From MAC genome	http://ciliates.org/	Sporadotrichida	Spirotrichea
Oxytricha trifallax	From MAC genome	http://ciliates.org/		
Halteria grandinella	From MAC genome	https://www.ncbi.nlm.nih.gov/		
Euplotes octocarinatus	From MAC genome	http://ciliates.ihb.ac.cn/	Euplotida	
Euplotes vannus	From MAC genome	http://ciliates.org/		
Euplotes aediculatus	From MAC genome	present work		

Table S2 Alignment statistics of orthologous gene pairs combined with the number of genes on the corresponding complete chromosomes among three *Euplotes* species

Query species	Subject species	1:1		1:N		N:1		N:N		Misaligned 1	Misaligned N
Euplotes aediculatus	*Euplotes octocarinatus*	8379	15194	3014	1104	2767	9769	1506	1016	752	129
Euplotes aediculatus	*Euplotes vannus*	5676	7652	1380	373	2166	5221	728	325		
Euplotes octocarinatus	*Euplotes aediculatus*	15259	8345	9826	2748	1110	3000	1032	1501	1216	50
Euplotes octocarinatus	*Euplotes vannus*	12461	9678	3565	492	1030	3610	402	295		
Euplotes vannus	*Euplotes aediculatus*	7846	5763	5343	2159	407	1438	347	723	9458	354
Euplotes vannus	*Euplotes octocarinatus*	9915	12688	3709	1041	518	3784	310	410		

query species: species of BLAST query sequences.

subject species: species of BLAST subject sequence.

1:1: orthologous gene pair between two species are all located on chromosomes containing a single gene.

1:N: orthologous gene pair between two species are located on chromosome containing a single gene and chromosome containing multiple genes, respectively.

N:1: orthologous gene pair between two species are located on chromosome containing multiple genes and chromosome containing a single gene, respectively.

N:N: orthologous gene pair between two species are all located on chromosomes containing multiple genes.

Misaligned 1: chromosome containing a single gene of query species that do not aligned with chromosome of the other two subject species.

Misaligned N: chromosome containing multiple genes of query species that do not aligned with chromosome of the other two subject species.

Table S3 The proportions of nanochromosomes with one gene in MAC genome for the class Spirotrichea

Species	The proportions of nanochromosomes with one gene (%)	Subclass	Average proportion of nanochromosomes with one gene (%)
Strombidium stylifer	73	Oligotrichia	73
Halteria grandinella	77	Hypotrichia	87
Oxytricha trifallax	81		
Stylonychia lemnae	82		
Pseudokeronopsis carnea	97		
Pseudokeronopsis flava	97		
Euplotes vannus	90	Euplotia	87
Euplotes octocarinatus	92		
Euplotes aediculatus	75		
Euplotes focardii	90		
